# Surfactin from *Bacillus subtilis* attenuates ambient air particulate matter-promoted human oral cancer cells metastatic potential

**DOI:** 10.7150/jca.48296

**Published:** 2020-08-18

**Authors:** Thi Thuy Tien Vo, Chiang-Wen Lee, Ching-Zong Wu, Ju-Fang Liu, Wei-Ning Lin, Yuh-Lien Chen, Lee-Fen Hsu, Ming-Horng Tsai, I-Ta Lee

**Affiliations:** 1 School of Dentistry, College of Oral Medicine, Taipei Medical University, Taipei, Taiwan.; 2 Department of Orthopaedic Surgery, Chang Gung Memorial Hospital, Puzi City, Chiayi County 61363, Taiwan.; 3 Department of Nursing, Division of Basic Medical Sciences, and Chronic Diseases and Health Promotion Research Center, and Research Center for Chinese Herbal Medicine, Chang Gung University of Science and Technology, Puzi City, Chiayi County 61363, Taiwan.; 4 Department of Safety Health and Environmental Engineering, Ming Chi University of Technology, New Taipei City 24301, Taiwan.; 5 College of Medicine, Chang Gung University, Guishan Dist., Taoyuan City 33303, Taiwan.; 6 School of Oral Hygiene, College of Oral Medicine, Taipei Medical University, Taipei, Taiwan.; 7 Graduate Institute of Biomedical and Pharmaceutical Science, Fu Jen Catholic University, New Taipei City, Taiwan.; 8 Department of Anatomy and Cell Biology, College of Medicine, National Taiwan University, Taipei, Taiwan.; 9 Department of Respiratory Care, Chang Gung University of Science and Technology, Puzi City, Chiayi County 613, Taiwan.; 10 Division of Neurosurgery, Department of Surgery, Chang Gung Memorial Hospital, Puzi City, Chiayi County 613, Taiwan.; 11 Department of Pediatrics, Division of Pediatric Hematology/Oncology and Neonatology, Yunlin Chang Gung Memorial Hospital, Yunlin, Taiwan.; 12 College of Medicine, Chang Gung University, Taoyuan, Taiwan.

**Keywords:** Surfactin, Invasion, Particulate matter, Oral cancer, Matrix metalloproteinase

## Abstract

Recently, many studies have indicated that ambient air particulate matter (PM) can increase the risk of oral cancer. The most common malignant tumor in the oral cavity is oral squamous cell carcinoma (OSCC). Usually, cancer cell migration/invasion is the most important cause of cancer mortality. Matrix metalloproteinase-2 (MMP-2) and MMP-9 have been shown to play important roles in regulating metastasis and the tumor microenvironment. Here, we studied the anti-cancer effects of surfactin, a cyclic lipopeptide generated by *Bacillus subtilis*, on cancer cell migration and invasion. Surfactin suppressed PM-promoted cell migration and invasion and colony formation of SCC4 and SCC25 human oral squamous cell carcinoma cell lines. We observed that PM induced MMP-2 and MMP-9 expression, which was inhibited by surfactin. Transfection with p65, p50, c-Jun, c-Fos, p85, p110, Akt, mammalian target of rapamycin (mTOR), or interleukin-6 (IL-6) siRNA markedly inhibited PM-induced MMP-2 and MMP-9 expression. Moreover, surfactin could reduce Akt, mTOR, p65, and c-Jun activation and IL-6 secretion induced by PM. Finally, we proved that transfection with Akt, p65, or c-Jun siRNA significantly inhibited PM-induced IL-6 release. Taken together, these results suggest that surfactin functions as a suppressor of PM-induced MMP2/9-dependent oral cancer cell migration and invasion by inhibiting the activation of phosphoinositide 3-kinase (PI3K)/Akt/mTOR and PI3K/Akt/nuclear factor-κB (NF-κB) and activator protein-1 (AP-1)/IL-6 signaling pathways.

## Introduction

In many parts of the world, new cases of oral cancer and deaths are increasing. Known risk factors include smoking, drinking, human papillomavirus (HPV), and betel quid chewing 1. It is also believed that exposure to heavy metals and emissions from petroleum and chemical plants is also related to the development of oral cancer, and it is well known that air pollution, especially ambient air particulate matter (PM), is harmful to the respiratory and cardiovascular system 2. The combined effects of household and ambient air pollution cause approximately 7 million premature deaths every year, mainly due to heart disease, stroke, lung cancer, chronic obstructive pulmonary disease, and acute respiratory infections leading to increased mortality 3, 4. The composition of PM is very complicated including nitrate, sulfate, ammonia, and so on. Compared to PM10, PM2.5 can cause greater harm to human health. PM2.5 generally penetrates the lung barrier and enters the blood system. Most studies in the past have explored the relationship between betel nuts, cigarettes, or alcohol and oral cancer, but few studies have studied the relationship between air pollution and oral cancer. The most common malignant tumor in the oral cavity is oral squamous cell carcinoma (OSCC). Moreover, cancer cell migration/invasion is the most important cause of cancer mortality 5. Matrix metalloproteinases (MMPs) belong to a family of zinc-dependent endopeptidases 6. Members of the MMP family include collagenase, gelatinase, stromalysin, stromelysin, and membrane-type MMP 6. Moreover, MMP-9 can mediate extracellular matrix (ECM) remodeling by cleaving many ECM proteins. MMP-2 or MMP-9 has been shown to play an important role in regulating metastasis including oral cancer 5, 7. PM2.5 also has been shown to induce MMP-2 and MMP-9 expression in keratinocytes 6. Therefore, reducing the expression of MMP-2 and MMP-9 or its upstream regulatory signaling pathways is essential for the treatment of malignant tumors. Up-regulation of MMP-2 and MMP-9 expression was mediated through various signaling pathways 8, 9. Here, we studied the novel pathways involved in PM-mediated MMP-2 and MMP-9 expression in the SCC4 and SCC25 human oral squamous cell carcinoma cell lines.

Surfactin is a bacterial cyclic lipopeptide generated by *Bacillus subtilis* 10. Surfactin has been shown to possess some properties including anti-cancer, anti-bacterial, and anti-viral activities 11. Even though surfactin has been regarded as a potential anti-cancer agent, its specific effects on cancer cells and the detailed mechanisms involved are still unknown. Park et al. indicated that surfactin reduced TPA-mediated breast cancer cell migration/invasion via the inhibition of MMP-9 levels 10. In addition, Wang et al. also proved that surfactin could promote apoptosis of HepG2 cells via the reactive oxygen species (ROS) signaling 12. Here, we studied the anti-cancer effects of surfactin on PM-induced human oral squamous cell carcinoma cell migration and invasion and the novel mechanisms underlying these processes. The present study proved that PM induces MMP-2/9-dependent cell migration and invasion via the phosphoinositide 3-kinase (PI3K)/Akt/mammalian target of rapamycin (mTOR) and PI3K/Akt/NF-κB and activator protein-1 (AP-1)/interleukin-6 (IL-6) signaling pathways. Moreover, surfactin can inhibit MMP-2/9 expression via inhibition of the activation of these two pathways induced by PM and then reduce cell migration and invasion.

## Methods

### Materials

We purchased anti-Akt, anti-phospho-Akt, anti-mTOR, anti-phospho-mTOR, anti-MMP-2, anti-MMP-9, anti-GAPDH, anti-tissue inhibitor of matrix metalloproteinase (TIMP)-1, anti-TIMP-2, anti-phospho-c-Jun, and anti-phospho-p65 antibodies from Santa Cruz Biotechnology Inc (Santa Cruz, CA, USA). Surfactin and urban PM (SRM 1648a) were purchased from Sigma (St. Louis, MO, USA).

### Cell culture

SCC4 and SCC25 human oral squamous cell carcinoma cell lines were kindly provided by Dr. J. F. Liu (School of Oral Hygiene, College of Oral Medicine, Taipei Medical University, Taipei, Taiwan). SCC4 and SCC25 cells were grown in DMEM/F12 supplemented with 10% FBS, 2 mM glutamine and 0.4 μg/ml hydrocortisone. Cells were maintained as monolayer cultures in a humidified atmosphere of 5% CO_2_ at 37°C.

### Cell viability

The cell viability of SCC4 and SCC25 cells in response to PM or surfactin was assessed using PrestoBlue Cell Viability Reagent (Invitrogen, CA, USA) according to the manufacturer's protocol.

### Western blot

Cells were incubated with PM under various experimental design conditions at 37°C. Western blot was then performed based on previously published literature in our laboratory 13. At last, membranes were incubated with the anti-Akt, anti-phospho-Akt, anti-mTOR, anti-phospho-mTOR, anti-MMP-2, anti-MMP-9, anti-GAPDH, anti-TIMP-1, anti-TIMP-2, anti-phospho-c-Jun, or anti-phospho-p65 antibody for 24 h and then incubated with the anti-mouse or anti-rabbit horseradish peroxidase antibody for 1 h. We used enhanced chemiluminescence (ECL) reagents to observe immunoreactive bands.

### Real-Time PCR

Total RNA was extracted by using TRIzol reagent. We further reverse-transcribed mRNA into cDNA and analyzed by real-time PCR using SYBR Green PCR reagents (Applied Biosystems, Branchburg, NJ, USA) based on previously published literature in our laboratory 14.

### Transient transfection with siRNAs

Human scrambled, MMP-2, MMP-9, p65, p50, c-Jun, c-Fos, p85, p110, Akt, mTOR, and IL-6 siRNAs were from Sigma (St. Louis, MO). Transient transfection of siRNAs was performed using a Lipofectamine 2000 Transfection Reagent according to the manufacturer's instructions.

### Analysis of luciferase reporter gene activity

Human MMP-2, MMP-9, AP-1, and NF-κB promoter-luciferase constructs were kindly provided by Dr. C. W. Lee (Department of Nursing, Chang Gung University of Science and Technology, Puzi City, Chiayi County, Taiwan). The luciferase activity was quantitatively assessed as previously described 15 using a luciferase assay system (Promega, Madison, Wis.). Firefly luciferase activities were standardized for β-gal activity.

### Gelatin zymography

Cells were seeded onto 6-well culture plates and made quiescent at confluence by incubation in serum-free DMEM/F12 for 24 h. Growth-arrested cells were treated with PM under various experimental design conditions at 37°C. The culture medium was collected and centrifuged at 10000 × *g* for 5 min at 4°C to remove cell debris. The expression of MMP-2 and MMP-9 was determined as previously published literature in our laboratory 15.

### Measurement of IL-6 secretion

Cells were incubated with PM under various experimental design conditions and then the media were collected. The levels of IL-6 were assayed by using an IL-6 ELISA kit (BioSource International, Camarillo, CA) according to the manufacturer's instructions.

### Migration assay

Cells were cultured in respective 10-cm cell culture dishes and grown for 80% confluence, then starved for 24 h by using serum-free DMEM/F12. Next, a sterile scalpel blade was manually utilized to generate identically wide scratches through the center of the cellular monolayer of both cell lines under aseptic conditions. The cell debris was washed once by PBS for totally removal. Serum-free DMEM/F12 with or without PM at concentration of 50 μg/cm^2^ was then added into designated dishes after 1 h preincubation with 10 μM surfactin containing DNA synthesis inhibitor hydroxyurea. The cellular migration from the cell wound boundary was monitored by taking pictures using digital camera under light microscope (Olympus, Japan) at baseline and 24 h time point. The number of migratory cells was counted based on the resulting 4 phase images of each time point, and the mean value was calculated. The data was obtained from independently triplicate experiments.

### Invasion assay

For cell invasion assay, BioCoat® Matrigel^TM^ Invasion Chambers with 8.0 μm PET Membrane in two 24 well inserts that simulate the cell invasion through the extracellular matrix were utilized following the manufacturer's instructions. SCC4 and SCC25 cells were re-suspended in serum-free DMEM/F12, then loaded onto respective Matrigel-coated cell culture inserts and incubated for 24 h. After incubation, the remaining cells on the upper side of the membrane were carefully removed using cotton swabs. The invaded cells that attached on the lower side of the membrane were fixed with 70% ethanol for 10 min and stained with 2% ethanol containing 0.2% crystal violet. The number of invaded cells was then enumerated in four randomly different observatory fields under light microscope with ×10 objective to calculate the average sum of cells that had invaded through the membrane.

### Chromatin immunoprecipitation (ChIP) assay

The protocol was modified based on previous study 10. Cells were cultured in respective 10-cm dishes at density of 2 × 10^7^ cells per dish. Next, 1% formaldehyde as a cross-linking agent was added into the medium in the cell culture dishes for 10 min at room temperature. The cross-linking reaction was quenched by using 0.125 M glycine. For chromatin fragmentation, the sample was aliquoted and subjected to sonication, followed by nuclease digestion with 10 U of MNase at 37°C for 15 min to produce chromatin at primarily mononucleosome size. Next, fragmented chromatin was bound to specific antibodies for 3 h at 4°C for immunoprecipitation. Protein-DNA complexes were recovered by using protein A agarose beads, then washed and eluated with elution buffer. The eluated complexes were incubated in 0.25 M NaCl at 65°C overnight to reverse the formaldehyde-caused-crosslinks, followed by digested with proteinase K for 2 h at 50°C to digest the protein. The immunoprecipitated DNAs were subsequently isolated and used for PCR experiments.

### Colony formation assay

After incubating SCC4 and SCC25 cells with PM with or without surfactin, these cells were seeded in complete media at a density of 2 × 10^4^ cells in 60-mm dishes containing a top layer of 0.7% agar and a bottom layer of 1% agar. The plates were incubated at 37°C for 14 days and then stained with 0.2% crystal violet. Colonies of greater than 20 cells were counted manually.

### Statistical analysis

We analyzed the data with the GraphPad Prism program (GraphPad, San Diego, CA, USA). Quantitative data were expressed as the mean±S.E.M. and analyzed with one-way ANOVA followed with Tukey's post-hoc test. We considered *P*<0.05 as a significant difference.

## Results

### The effects of PM and surfactin on the cell viability of SCC4 and SCC25 cells

**Figure 1:**
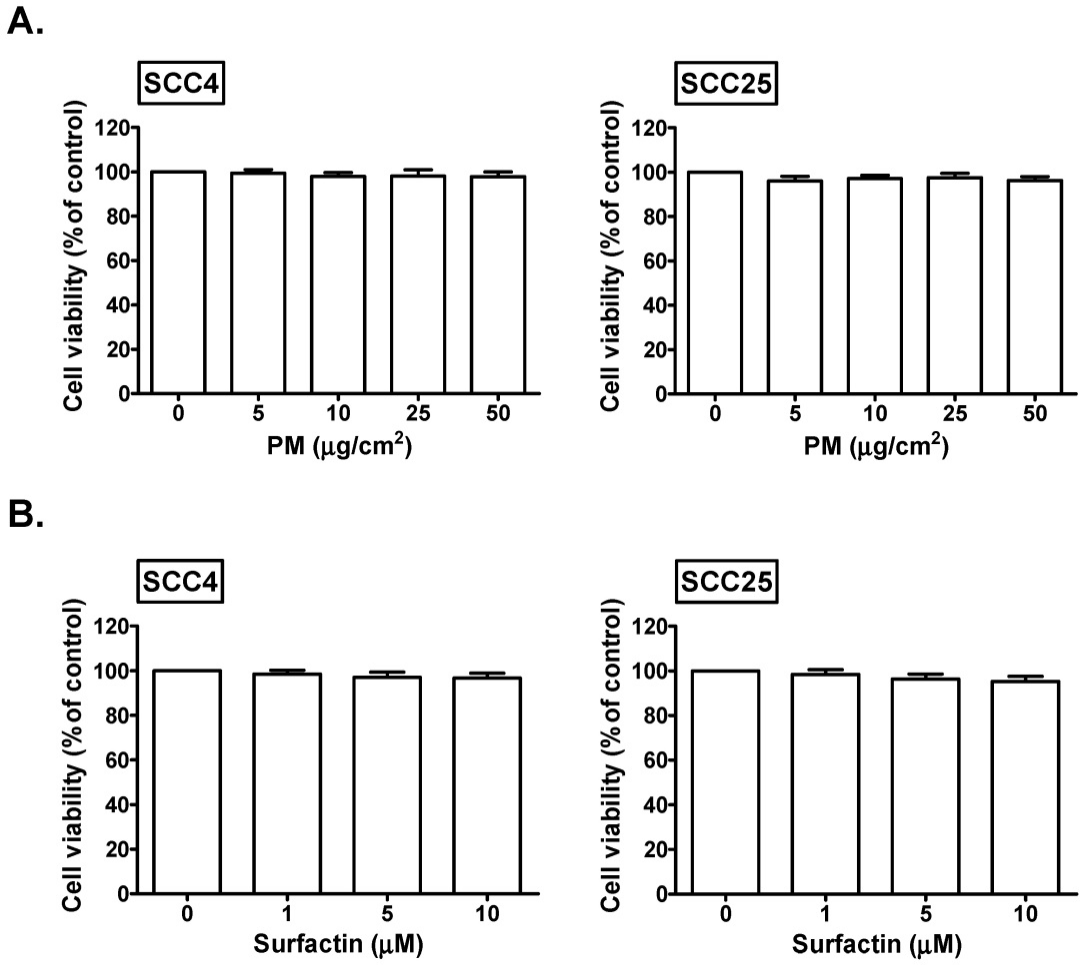
** The effects of PM and surfactin on SCC4 and SCC25 cell viability.** Cells were treated with the indicated concentrations of (**A**) PM or (**B**) surfactin for 24 h. The cell viabilities were then measured.

We explored the cell viability of SCC4 and SCC25 cells in response to PM and surfactin. We found that 5, 10, 25, and 50 μg/cm^2^ PM had no significant effects on the cell viability of SCC4 and SCC25 cells (Fig. 1A). In addition, the cell viability of SCC4 and SCC25 cells was not affected in response to 1, 5, and 10 μM surfactin (Fig. 1B). Therefore, 50 μg/cm^2^ PM and 10 μM surfactin were applied in all the subsequent experiments.

### Surfactin reduces PM-induced migration, invasion, and colony formation of SCC4 and SCC25 cells

**Figure 2:**
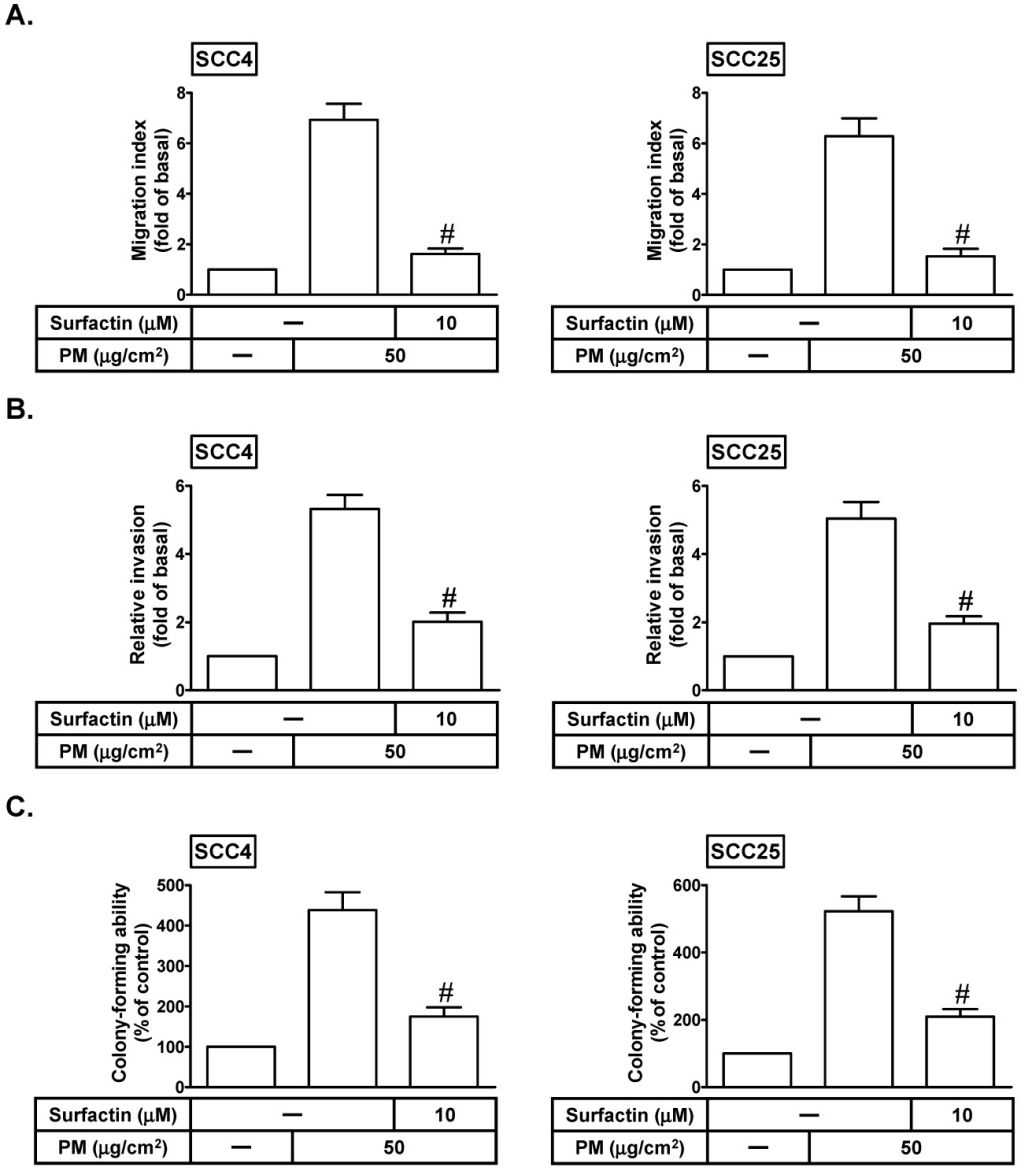
** Surfactin decreases PM-induced migration, invasion, and colony-forming ability of SCC4 and SCC25 cells.** Cells were pretreated with surfactin for 1 h and then incubated with PM for 24 h. The cell migration assay and matrigel invasion assay were performed and then the (**A**) migrating cells and (**B**) invaded cells were counted. (**C**) Cells were cultured in soft agar gel with PM for 14 days in the absence or presence of surfactin. The colonies were counted. Data are expressed as mean±S.E.M. of three independent experiments. #*P* < 0.01, as compared with the cells exposed to PM alone.

Cancer cell migration/invasion is the most important cause of cancer mortality 5. Surfactin has been shown to possess some properties including anti-cancer, anti-bacterial, and anti-viral activities 11. We investigated whether surfactin could inhibit PM-induced cell migration and invasion. SCC4 and SCC25 cells were treated with PM for 24 h in the presence or absence of surfactin. As shown in Figs. 2A and B, PM could induce cell migration and invasion, which was inhibited by surfactin. The process of tumor metastasis is very complicated including the proteolytic digestion of ECM, cell migration to the circulatory system and colonization at the site of metastasis 16. Thus, we observed the effects of surfactin on colony-forming ability in response to PM in SCC4 and SCC25 cells. As shown in Fig. 2C, we proved that surfactin could suppress PM-mediated colony-forming ability in these cells.

### Surfactin inhibits PM-induced MMP-2 and MMP-9 expression and enzyme activity

**Figure 3:**
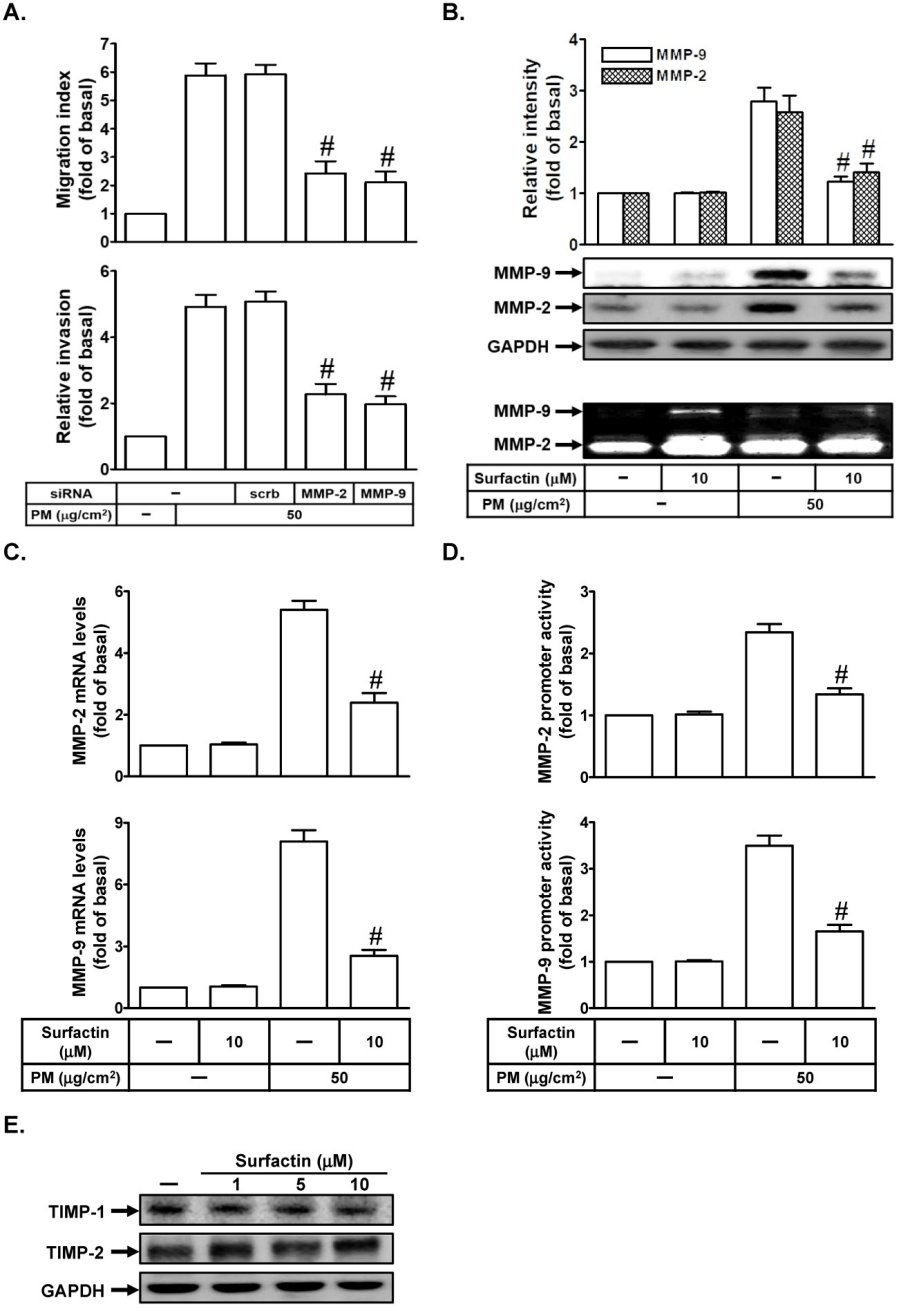
** Surfactin suppresses MMP-2 and MMP-9 expression and enzyme activity induced by PM.** (**A**) SCC4 cells were transfected with siRNA of scrambled (scrb), MMP-2, or MMP-9 and then treated with PM for 24 h. The cell migration assay and matrigel invasion assay were performed and then the migrating cells and invaded cells were counted. (**B**) SCC4 cells were pretreated with surfactin for 1 h and then incubated with PM for 24 h. Culture supernatant was analyzed by gelatin zymography. The cellular extract was analyzed by Western blot. SCC4 cells were pretreated with surfactin for 1 h and then incubated with PM for 6 h. The (**C**) mRNA levels and (**D**) promoter activity of MMP-2 and MMP-9 were determined. (**E**) SCC4 cells were treated with the indicated concentrations of surfactin for 24 h. The protein levels of TIMP-1 and TIMP-2 were determined by Western blot. Data are expressed as mean±S.E.M. of three independent experiments. #*P* < 0.01, as compared with the cells exposed to PM + scrambled siRNA (A). #*P* < 0.01, as compared with the cells exposed to PM alone (B-D).

MMP-9 or MMP-2 has been shown to play an important role in regulating metastasis 17. We studied whether MMP-2 and MMP-9 were involved in PM-induced cell migration and invasion. As shown in Fig. 3A, we proved that transfection with MMP-2 or MMP-9 siRNA markedly inhibited PM-induced cell migration and invasion in SCC4 cells. We further investigated whether surfactin could reduce PM-induced MMP-9 and MMP-2 expression and activity. As shown in Figs. 3B-D, we found that PM could enhance MMP-2 and MMP-9 protein expression, enzyme activity, mRNA levels, or promoter activity, which was inhibited by surfactin in SCC4 cells. Surfactin had similar inhibitory effects on MMP-9 expression induced by PM in SCC25 cells (data not shown). TIMP is the endogenous inhibitor of most secreted MMPs 18. Finally, we studied the effects of surfactin on TIMP-1 and TIMP-2 expression. As shown in Fig. 3E, surfactin had no effects on TIMP-1 and TIMP-2 protein levels in SCC4 cells.

### Surfactin suppresses PM-induced MMP-2 and MMP-9 expression via inhibition of the activation of NF-κB and AP-1

**Figure 4:**
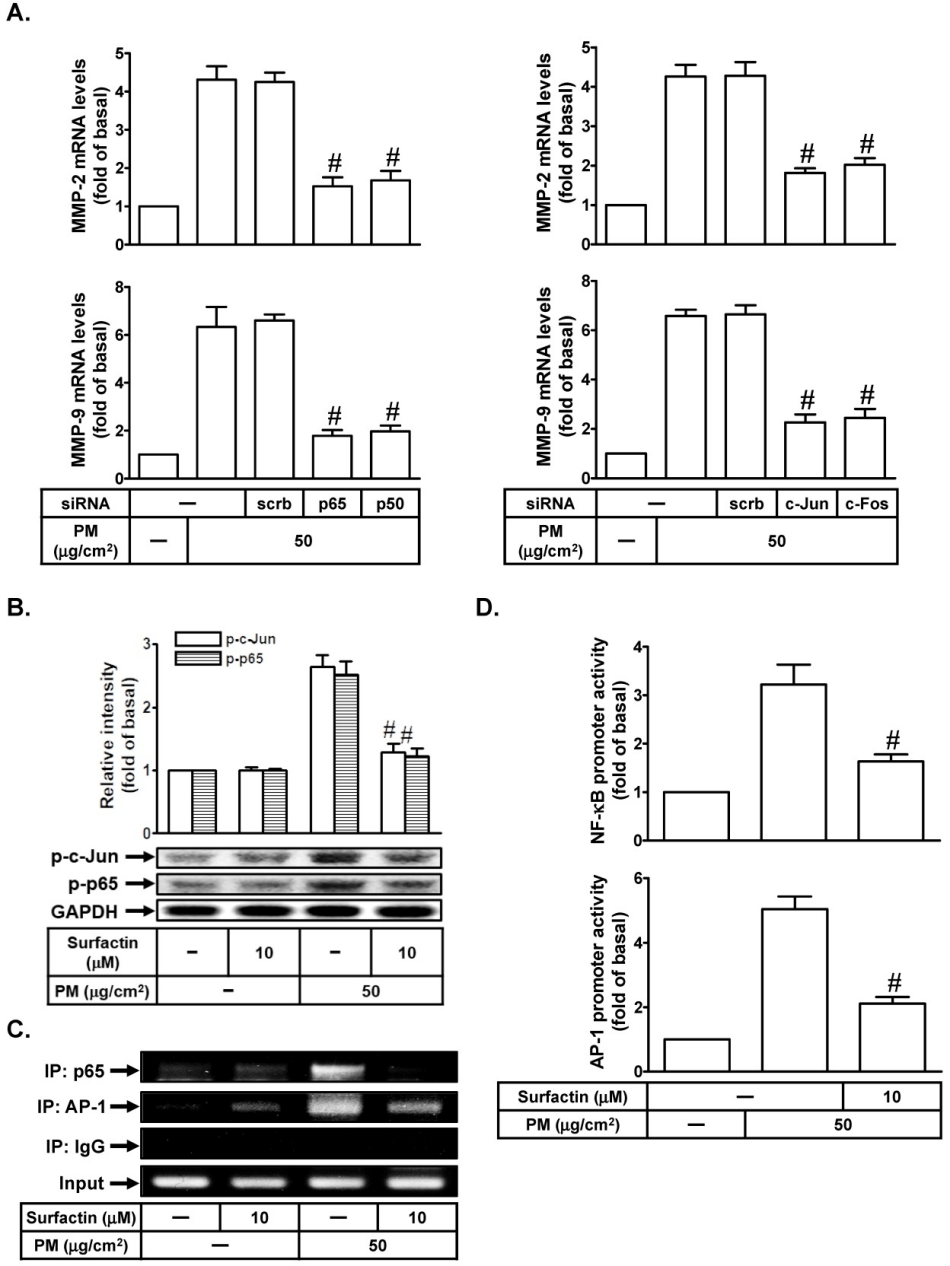
** Surfactin suppresses PM-induced NF-κB and AP-1 activation.** (**A**) SCC4 cells were transfected with siRNA of scrambled (scrb), p65, p50, c-Jun, or c-Fos and then treated with PM for 6 h. The mRNA levels of MMP-2 and MMP-9 were determined. (**B**) SCC4 cells were pretreated with surfactin for 1 h and then incubated with PM for 1 h. The protein levels of phospho-c-Jun (p-c-Jun) and phospho-p65 (p-p65) were determined by Western blot. (**C**) SCC4 cells were pretreated with surfactin for 1 h and then incubated with PM for 4 h. A ChIP assay was performed with anti-NF-κB p65 or anti-AP-1 antibodies. (**D**) SCC4 cells were pretreated with surfactin for 1 h and then incubated with PM for 1 h. The promoter activity of NF-κB and AP-1 were determined. Data are expressed as mean±S.E.M. of three independent experiments. #*P* < 0.01, as compared with the cells exposed to PM + scrambled siRNA (A). #*P* < 0.01, as compared with the cells exposed to PM alone (B, D).

Up-regulation of MMP-2 and MMP-9 expression was mediated through various signaling pathways including AP-1 and NF-κB 19, 20. We studied whether AP-1 and NF-κB were involved in PM-induced MMP-2 and MMP-9 expression in SCC4 cells. As shown in Fig. 4A, transfection with p65, p50, c-Jun, or c-Fos siRNA markedly reduced PM-mediated MMP-2 and MMP-9 mRNA levels in SCC4 cells. We further investigated whether surfactin could inhibit PM-induced AP-1 and NF-κB activation. As shown in Fig. 4B, we showed that PM could induce c-Jun and p65 phosphorylation, which was reduced by surfactin in SCC4 cells. Surfactin had similar inhibitory effects on c-Jun and p65 phosphorylation induced by PM in SCC25 cells (data not shown). We used a ChIP assay to observe the effects of surfactin on the binding activities of NF-κB and AP-1 with the MMP-9 promoter. As shown in Fig. 4C, we proved that PM could increase the *in vivo* binding of NF-κB and AP-1 to the MMP-9 promoter, which was reduced by surfactin. Finally, we showed that PM enhanced NF-κB and AP-1 promoter activity, which was also inhibited by surfactin (Fig. 4D).

### Surfactin decreases PM-enhanced MMP-2 and MMP-9 expression through the inhibition of activation of PI3K/Akt/mTOR signaling pathway

**Figure 5:**
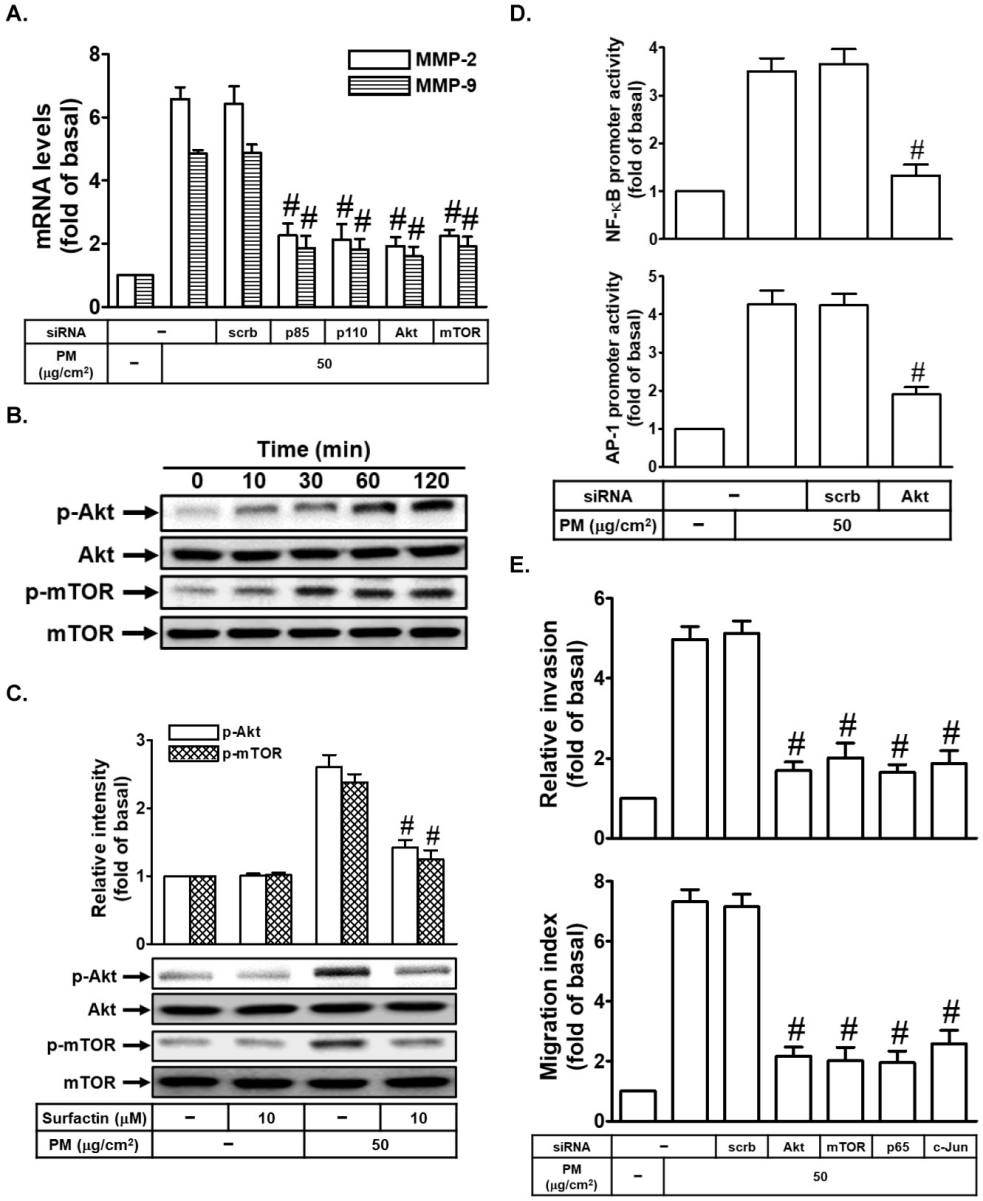
** Surfactin decreases PM-regulated PI3K/Akt/mTOR signaling pathway activation.** (**A**) SCC4 cells were transfected with siRNA of scrambled (scrb), p85, p110, Akt, or mTOR and then treated with PM for 6 h. The mRNA levels of MMP-2 and MMP-9 were determined. (**B**) SCC4 cells were treated with 50 μg/cm^2^ PM for the indicated times and then the protein levels of phospho-Akt and phospho-mTOR were determined by Western blot. (**C**) SCC4 cells were pretreated with surfactin for 1 h and then incubated with PM for 1 h. The protein levels of phospho-Akt and phospho-mTOR were determined. (**D**) SCC4 cells were transfected with siRNA of scrambled (scrb) or Akt and then treated with PM for 1 h. The promoter activity of NF-κB and AP-1 were determined. (**E**) SCC4 cells were transfected with siRNA of scrambled (scrb), Akt, mTOR, p65, or c-Jun and then treated with PM for 24 h. The cell migration assay and matrigel invasion assay were performed and then the migrating cells and invaded cells were counted. Data are expressed as mean±S.E.M. of three independent experiments. #*P* < 0.01, as compared with the cells exposed to PM + scrambled siRNA (A, D, E). #*P* < 0.01, as compared with the cells exposed to PM alone (C).

The PI3K/Akt/mTOR signaling is a frequently hyperactivated pathway in cancer and is critical for the growth and survival of tumor cells 21. We also studied whether the PI3K/Akt/mTOR signaling pathway was involved in PM-induced cell migration and invasion. At first, we proved that transfection with p85, p110, Akt, or mTOR siRNA markedly inhibited PM-induced MMP-2 and MMP-9 mRNA levels in SCC4 cells (Fig. 5A). Moreover, we also found that PM could induce Akt and mTOR phosphorylation in SCC4 cells (Fig. 5B). We then studied whether surfactin could inhibit Akt and mTOR activation induced by PM. As shown in Fig. 5C, we found that surfactin significantly reduced PM-caused Akt and mTOR activation in SCC4 cells. Surfactin had similar inhibitory effects on Akt and mTOR phosphorylation induced by PM in SCC25 cells (data not shown). AP-1 and NF-κB activation was regulated through many signaling pathways, such as PI3K/Akt 22, 23. In our study, we proved that transfection with Akt siRNA inhibited PM-induced NF-κB and AP-1 promoter activity in SCC4 cells (Fig. 5D). Finally, we showed that transfection with Akt, mTOR, p65, or c-Jun siRNA reduced PM-promoted cell migration and invasion (Fig. 5E).

### IL-6 is involved in PM-induced MMP-2 and MMP-9 expression

**Figure 6:**
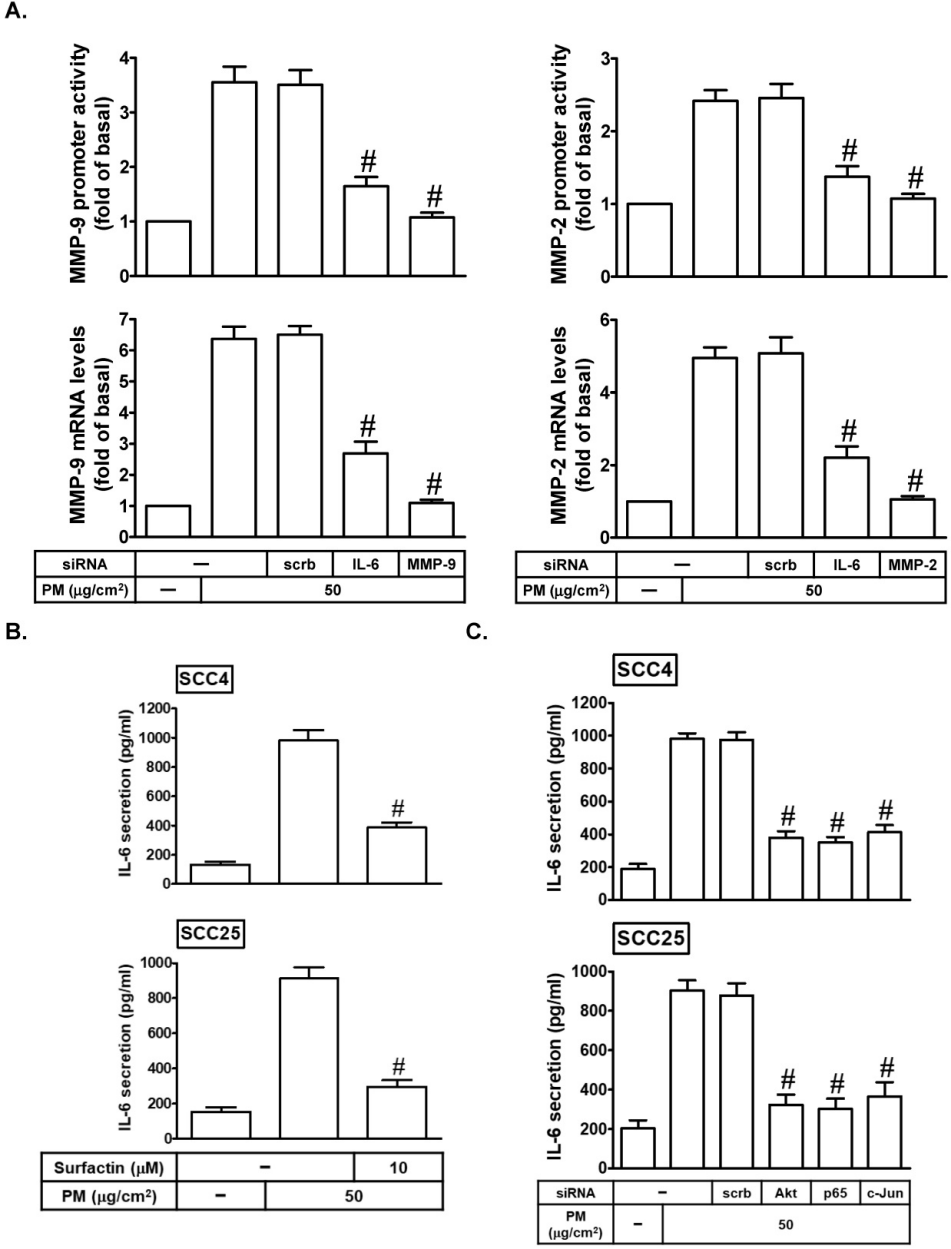
** PM induces MMP-2 and MMP-9 expression via IL-6.** (**A**) SCC4 cells were transfected with siRNA of scrambled (scrb), IL-6, MMP-2, or MMP-9 and then treated with PM for 6 h. The mRNA levels and promoter activity of MMP-2 and MMP-9 were determined. (**B**) SCC4 cells were pretreated with surfactin for 1 h and then incubated with PM for 24 h. The secretion of IL-6 was measured. (**C**) SCC4 cells were transfected with siRNA of scrambled (scrb), Akt, p65, or c-Jun and then treated with PM for 24 h. The secretion of IL-6 was measured. Data are expressed as mean±S.E.M. of three independent experiments. #*P* < 0.01, as compared with the cells exposed to PM + scrambled siRNA (A, C). #*P* < 0.01, as compared with the cells exposed to PM alone (B).

Many reports indicated that IL-6 can promote tumor growth and metastasis 24, 25. IL-6 also has been shown to regulate MMP-9 expression 26. Here, we found that transfection with IL-6 siRNA could markedly inhibit PM-induced MMP-2 and MMP-9 mRNA expression and promoter activity (Fig. 6A). On the other hand, we studied whether surfactin could reduce PM-induced IL-6 release in SCC4 and SCC25 cells. As shown in Fig. 6B, we proved that PM could enhance IL-6 secretion, which was reduced by surfactin in these cells. Finally, we investigated the pathways involved in PM-induced IL-6 release in SCC4 and SCC25 cells. As shown in Fig. 6C, we showed that transfection with siRNA of Akt, p65, or c-Jun significantly inhibited IL-6 secretion in response to PM. Therefore, these data suggest that PM can induce MMP-2 and MMP-9 expression via the PI3K/Akt/NF-κB and AP-1/IL-6 pathway, which was inhibited by surfactin.

## Discussion

PM exposure causes various inflammatory diseases. Chu et al. proved that Taiwanese men exposed to higher concentrations of PM2.5 have an increased risk of oral cancer 2. The seventh most common cancer in the world is OSCC 27. A very important step in tumor metastasis is that cancer cells invade the surrounding tissues and vasculature. However, the chemotactic migration of cancer cells is required in this process, which is controlled by protrusive activity of the cell membrane and its attachment to the ECM 28. MMP-2 and MMP-9 expression is known to promote the migration/invasion and metastasis of cancer cells 29. Surfactin has been shown to suppress cancer progression by cell cycle arrest, apoptosis, growth inhibition, and metastasis arrest 30. The potential mechanisms by which surfactin inhibits oral cancer cell migration and invasion in response to PM still unclear. Here, we proved that PM induces MMP-2/9-dependent cell migration and invasion via the PI3K/Akt/mTOR and PI3K/Akt/NF-κB and AP-1/IL-6 signaling pathways. Moreover, surfactin can inhibit MMP-2/9 expression via inhibition of the activation of these two pathways induced by PM and then reduce cell migration and invasion.

Most studies have explored the relationship between betel nuts, cigarettes, or alcohol and oral cancer, but few studies have studied the relationship between air pollution and oral cancer. PM is typically a representative indicator of air pollution 2, 4. Compared to PM10, PM2.5 can cause greater harm to human health 4. PM2.5 generally penetrates the lung barrier and enters the blood system. Zhang and Li indicated that PM2.5 could induce the cell proliferation, migration, and invasion of human hepatocellular carcinoma (HCC) cell line SMMC-7721 31. This is confirmed by our observation that PM could induce cell migration and invasion and colony-forming ability of SCC4 and SCC25 cells. *Bacillus subtilis* generates the cyclic lipopeptide surfactin. Its heptapeptide head has two negatively charged amino acid residues, and its tail consists of fatty acid residues 30. Surfactin has been shown to possess some properties including anti-cancer, anti-bacterial, and anti-viral activities 11. Moreover, we found that surfactin has the anti-oral cancer effects by our observation that surfactin inhibited PM-induced cell migration and invasion and colony-forming ability of SCC4 and SCC25 cells.

Degradation of the ECM is an important step in tumor cell invasion. Although various proteases are involved in ECM degradation, MMPs, a family of zinc and calcium-dependent proteolytic enzymes, digest many components of ECM 32. MMPs are important to cell migration/invasion and metastasis 32. At least more than 20 different MMPs work on multiple substrates including collagen types I, II, III, IV and stromyelin 32, 33. Among these MMPs, gelatinases, especially MMP-2 and MMP-9, are considered to play an important role in the degradation of type IV collagen and gelatin. The release of MMP-2 and MMP-9 is increased in various types of cancers including oral cancer and their increased levels are associated with poor prognosis 33. In the present study, we proved that PM could induce MMP-2 and MMP-9 activity and expression in SCC4 and SCC25 cells. On the other hand, PM2.5 has been shown to induce MMP-2 and MMP-9 expression in human keratinocytes and cause skin aging 6. Indeed, we also found that the levels of MMP-2 and MMP-9 in oral cancer cells or breast cancer cells are higher than in human gingival fibroblasts under the stimulation of PM (Supplementary Fig. 1). Moreover, surfactin can markedly reduce PM-induced MMP-2 and MMP-9 mRNA levels in these cells. These results suggest that in addition to inflammation, MMP-2 and MMP-9 may also contribute to the migration and invasion of cancer cells. Moreover, surfactin can inhibit PM-induced cell migration and invasion via inhibition of MMP-2 and MMP-9 expression in various cancer cells including oral cancer cells and breast cancer cells. TIMP is the endogenous inhibitor of most secreted MMPs 18. In our study, we proved that surfactin could not inhibit cell migration and invasion through induction of these endogenous inhibitors including TIMP-1 and TIMP-2. Thus, we suggest that surfactin has the anti-cancer effects by directly inhibiting MMP-2 and MMP-9 levels in response to PM.

Up-regulation of MMP-2 and MMP-9 expression was mediated through various signaling pathways including AP-1 and NF-κB 19, 20. NF-κB is the main mediator and can regulate the crosstalk between inflammation and cancer at multiple levels 34. AP-1 is composed of a Jun-Jun homodimer or a Jun-Fos heterodimer 35. AP-1 is involved in the control of many cancer cells 35. The PI3K/Akt/mTOR signaling is a frequently hyperactivated pathway in cancer and is critical for the growth and survival of tumor cells 21. AP-1 and NF-κB activation was regulated via many signaling pathways, such as Akt 22, 23. We proved that PM induced MMP-2 and MMP-9 expression via these signaling pathways by using siRNA of p65, c-Jun, p50, c-Fos, p85, p110, Akt, or mTOR. PM also caused p65, c-Jun, Akt, and mTOR activation in these cells. However, surfactin could inhibit MMP-2 and MMP-9-dependnet cell migration/invasion via inhibition of the activation of NF-κB, AP-1, Akt, and mTOR in response to PM. Although many studies have showed that surfactin has anti-cancer function, our research team first pointed out that surfactin can inhibit oral cancer migration and invasion induced by PM. In the future, we will investigate other novel signaling pathways involved in surfactin-inhibited MMP-2 and MMP-9 expression and activity in response to PM.

IL-6 is one of the cytokines in the tumour microenvironment. In addition, IL-6 is a critical factor which is detected at high levels and shown to be deregulated in cancer. Many reports indicated that IL-6 can promote tumor growth and metastasis 24, 25. IL-6 also has been shown to regulate MMP-9 expression 26. This is confirmed by our observation that IL-6 siRNA suppressed PM-induced MMP-2 and MMP-9 expression. As we expected, surfactin decreased IL-6 secretion induced by PM. In the future, we will investigate other cytokines involved in PM-caused cell migration and invasion. However, surfactin can achieve anti-cancer effects by inhibiting these cytokines induced by PM.

**Figure 7:**
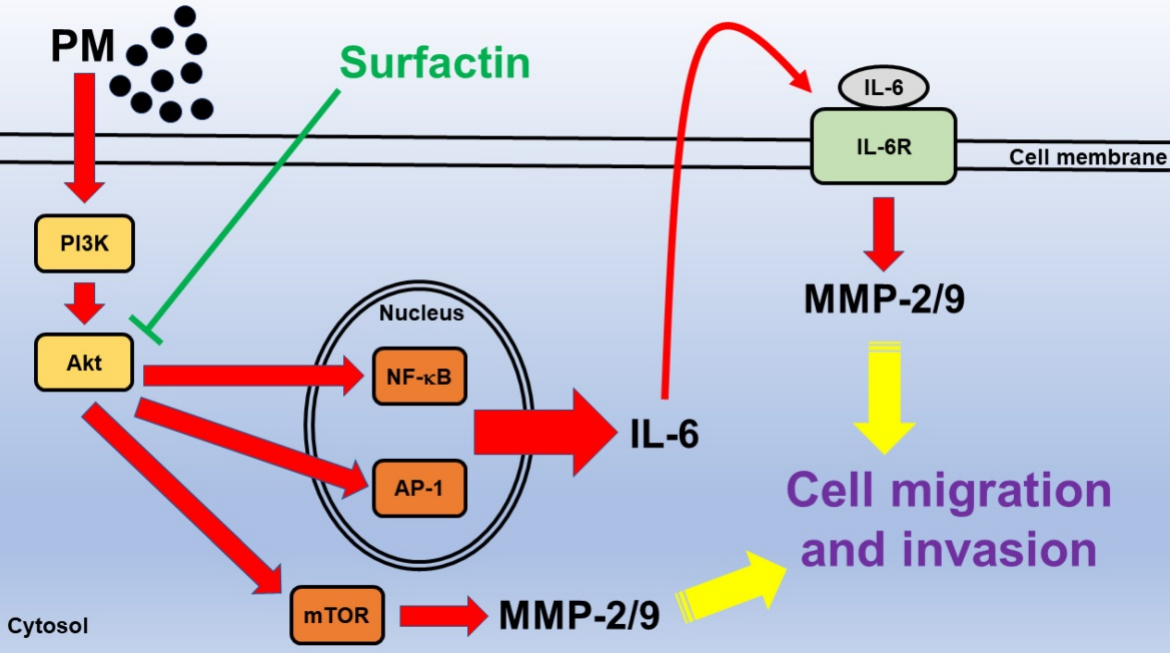
** Schematic diagram illustrating the signaling pathway involved in surfactin-inhibited PM-induced human oral squamous cell carcinoma cell line migration and invasion.** PM induces MMP-2/9-dependent cell migration and invasion via the PI3K/Akt/mTOR and PI3K/Akt/NF-κB and AP-1/IL-6 signaling pathways. Moreover, surfactin from *Bacillus subtilis* can inhibit MMP-2/9 expression via inhibition of the activation of these two pathways induced by PM and then reduce cell migration and invasion.

In Fig. 7, we prove that in SCC4 and SCC25 cells, PM induces MMP-2/9-dependent cell migration and invasion via the PI3K/Akt/mTOR and PI3K/Akt/NF-κB and AP-1/IL-6 signaling pathways. Surfactin from *Bacillus subtilis* can inhibit MMP-2/9 expression via inhibition of the activation of these two pathways induced by PM and then reduce cell migration and invasion. These experimental data also give us a new direction for the prevention and treatment of oral cancer.

## Supplementary Material

Supplementary figure S1.
