# Supplementary material for: Surfactin from Bacillus subtilis attenuates ambient air particulate matter-promoted human oral cancer cells metastatic potential
Source: J Cancer. 2020 Aug 18;11(20):6038–49. doi: 10.7150/jca.48296 (PMC7477423; doi:10.7150/jca.48296)
Supplement: Supplementary file 1 — Supplementary figure S1. [file jcav11p6038s1.pdf]

Supplementary Fig. 1

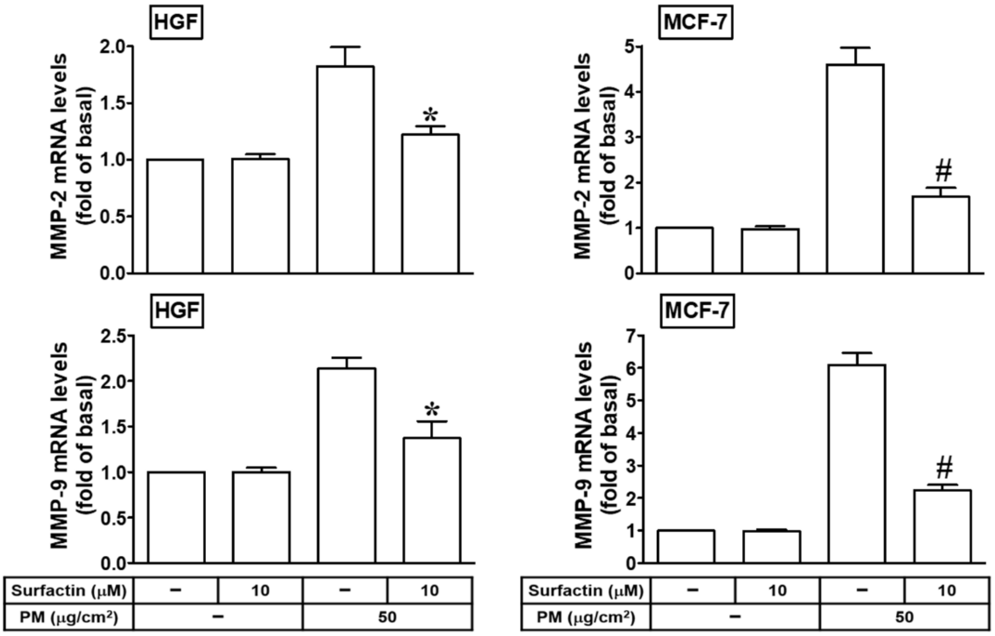

Supplementary Figure 1. The effects of surfactin on PM-mediated MMP-2 and MMP-9 expression in human gingival fibroblasts and human breast cancer cells. Cells were pretreated with surfactin for 1 h and then incubated with PM for 6 h. The mRNA levels of MMP-2 and MMP-9 were determined.
